# Gender difference of metabolic syndrome and its association with dietary diversity at different ages

**DOI:** 10.18632/oncotarget.20625

**Published:** 2017-09-02

**Authors:** Xu Tian, Xiaohui Xu, Kai Zhang, Hui Wang

**Affiliations:** ^1^ College of Economics and Management, China Center for Food Security Studies, Nanjing Agricultural University, Nanjing, Jiangsu, China; ^2^ Department of Epidemiology and Biostatistics, School of Public Health, Nanjing Medical University, Nanjing, Jiangsu, China; ^3^ Pancreatic Center and Department of General Surgery, The First Affiliated Hospital of Nanjing Medical University, Nanjing,Nanjing, Jiangsu, China

**Keywords:** age, sex, dietary diversity score, metabolic syndrome, Pathology Section

## Abstract

**Background:**

Previous research indicated that dietary diversity had favorable association with metabolic syndrome (MetS), and it has not been investigated in China.

**Methods:**

Adults (aged 18+) with complete dietary and biochemical data were collected from 2009 China Health and Nutrition Survey (*n*=4308). Dietary diversity was measured by modified Dietary Diversity Score (DDS). MetS was defined by the harmonized criteria. The association between DDS and MetS was investigated by multivariable adjusted logistic regression.

**Results:**

An inverse-U shape relationship between MetS risk and age was detected for both genders, and female were more vulnerable than male at old times. More diversified diet decreased the risk of MetS for young female (≥18 & ≤45), similar trends were detected in serum TGs, abdominal adiposity, blood pressure, and fasting blood glucose (all *P*<0.05). However, this association reversed for old female (>60) and male adults (>45&≤60). Greater DDS was associated with higher serum TGs, and lower HDL-C level for male adults, higher blood pressure for old men, but lower blood pressure and fasting blood glucose in young men (all *P*<0.05).

**Conclusion:**

Male adults and old female had the highest risk of getting MetS. More diversified diet decreased MetS risk for young female, but increased the risk for male adults and old female.

## INTRODUCTION

Metabolic syndrome (MetS) is a complex of anthropometric and biochemical marker aberrations including abdominal obesity, insulin resistance, dyslipidemia and high blood pressure [1] . MetS was considered as a major risk factor of cardiovascular disease (CVD) and type 2 diabetes (T2D) [2]. CVD is the leading cause of death worldwide and accounts for 30 percent of global deaths in 2008 according to the World Health Organization (WHO)’s report [3]. Meanwhile, approximately 415 million people had the T2D worldwide, and it will rise to 642 million [4]. Approximately one in five adults in China suffered from CVD and the proportion is expected to increase in the next few decades [5]. Moreover, one study estimated that prevalence of diabetes was around 12% in 2013 in China, of which 50% had prediabetes [6]. These two non-communicable diseases are lifelong and impair the quality of life.

Diet is a key melleable risk factor for multiple chronic diseases [7]. Globally, approximately 5% excess mortality can be attributed to inadequate consumption of fruits and vegetables [7]. Several articles highlight the importance of a healthy balanced diet, particularly the diversity of food consumption [8]. A more diversified diet, if it is characterized by increasing variety within nutrient-dense foods and displacing intake of energy-dense foods, could become a long-term strategy of a healthful diet and further contributes to reducing adiposity and cardio-metabolic health [9, 10]. For instance, greater intake of fruits, vegetables and dairy products is associated with higher intake of fiber, vitamin C and calcium [11, 12], which are believed to play a protective function in pathogenesis of CVD and T2D [13-16]. However, greater dietary variety could also lead to overeating due to increasing enjoyability of new individual food item [9, 17-19], which might contribute to raising prevalence of obesity if more high-calorie-density foods are consumed [20].

Previous studies found a negative association between dietary diversity and MetS in Iran, Korea, and Brazil [21-23]. Similarly, more diversified diet is found to be associated with a lower risk of being MetS in the U.S., using the U.S. Healthy Food Diversity (HFD) index [24]. On the contrary, dietary diversity was observed to be associated with higher risk of obesity for Chinese men with the adoption of Dietary Diversity Score (DDS) [20] . DDS is a widely used indicator measuring the diversity of diet, which is easy to calculate and represents the adequacy ratio of some nutrients and overall quality of diet [25]. Along with the successful economic growth in the past four decades, China experienced rapid development of food market; consequently, food availability and diversity increased substantially [19, 26, 27]. However, current literatures have not explored the association between dietary diversity and MetS in China. We thus investigated the association between dietary diversity and MetS using Chinese Health and Nutrition Survey (CHNS) data.

## RESULTS

### Population characteristics.

Table 1 presented the mean and S.D. of socio-economic variables at each tertile of DDS. Female adults in the second & third DDS tertile were younger and richer, better educated, had lower physical activity, smoke less but drank more, and were likely to come from urban area (all p<0.05). Similarly, male adults at higher DDS tertiles were also younger, richer, better educated, had lower physical activity but higher BMI, and were more likely to come from urban area (all p trends <0.05).

**Table 1:** Characteristics of participants by DDS tertiles^1^ and sexes

	Female				Male			
	Tertile 1	Tertile 2	Tertile 3	p trend^2^	Tertile 1	Tertile 2	Tertile 3	p trend
Number	700	1026	622		593	905	462	
Age(y)	50.8 ±14.6	47.8 ± 14.2	47.9 ± 14.2	<0.001	50.5 ± 15.1	49.3 ± 14.7	48.7 ± 15.8	0.047
ln(income)^3^	9.6 ±1.6	10.0 ± 1.4	10.5 ± 0.9	<0.001	9.8 ± 1.4	10.0 ± 1.6	10.4 ± 1.07	<0.001
Educational level(%)^4^								
primary	59	46	33	<0.001	40	33	22	<0.001
middle	40	49	55	0.001	57	62	64	0.237
high	1	5	11	<0.001	3	5	13	<0.001
Physical activity(%)^5^								
light	40	54	75	<0.001	31	42	59	<0.001
middle	13	11	12	0.612	14	18	20	0.030
heavy	47	35	13	<0.001	55	40	21	<0.001
Smoking(%)	4	3	2	0.033	59	57	56	0.607
Drinking(%)	6	9	11	0.003	58	64	66	0.186
Rural (%)	82	73	45	<0.001	82	73	50	<0.001
North(%)	47	36	48	0.914	45	33	50	0.491
BMI	23.2±3.6	23.2±3.4	23.1±3.3	0.776	22.7±3.2	23.1±3.3	23.4±3.4	0.001
BMI≥28(%)	11	9	8	0.138	5	7	8	0.110

1. Tertile cut-points of DDS are as follows: 1st, ≤3; 2nd, 4; 3rd, 5-6. Continuous variables are presented as mean ± SD and categorical variables are presented as percentages.

2. P trend was analyzed by chi-square trends analysis.

3. ln(income) means logarithm of income per capita.

4. Education level (primary school education, middle school education, or above high school education).

5. Physical activity (according to the occupation type, ranging from 1 to 5; this classification conforms to the original data from the CHNS: 1 = very light physical activity, working in a sitting position [e.g., office worker or watch repairer]; 2 = light physical activity, working in a standing position [e.g., sales person or teacher]; 3 = moderate physical activity [e.g., student or driver]; 4 = heavy physical activity [e.g., farmer or dancer]; and 5 = very heavy physical activity [e.g., loader, logger, or miner]; 1 and 2 are classified as light activity, 3 are classified as moderate activity, 4 and 5 are classified as heavy activity).

### Dietary information and Biochemical marker, MetS and its components

Table 2 presented the mean and S.D. of dietary and biochemical markers for male and female at each DDS tertile. Adults (both male and female) at higher DDS tertiles were found to have higher energy intake (mainly caused by the higher intake of protein and fat), but lower carbohydrate intake, more consumption of fruits, meat, dairy and bean, but less consumption of grain (all p trends <0.05). To be note, the difference between second and third tertile was not significant. Moreover, higher waist circumference was observed at more diversified diet for male, while an inverse relationship was found for female. However, the differences in waist circumference among different tertiles were negligible. In addition, lower systolic blood pressure was associated with upper categorical of DDS for female, while no significant trend was detected for other biomarkers over different DDS tertiles. Intriguingly, the triglyceride and fasting blood glucose increased significantly with greater DDS for men, while the HDL-C decreased significantly at greater DDS level. We also found increasing percentage of MetS and low HDL-C at higher tertiles of DDS for men.

**Table 2:** Dietary intake and metabolic risk factors of participants by DDS tertiles^1^ and sexes

	Female				Male			
	Tertile 1	Tertile 2	Tertile 3	p trend^2^	Tertile 1	Tertile 2	Tertile 3	p trend
Number	700	1026	622		593	905	462	
Nutrients								
Total energy (kcal/day)	1848±554	2007±578	1967±525	<0.001	2182±622	2378±655	2344±626	<0.001
Carbonhydrate (g/day)	280±97	274±95	257±83	<0.001	330±112	320±101	302±92	<0.001
Protein (g/day)	51±16	63±20	66±18	<0.001	60±18	74±22	77±23	<0.001
Fat (g/day)	58±30	73±32	75±29	<0.001	65±32	84±38	89±38	<0.001
Foods(g/day)								
Grain	369±143	340±131	304±121	<0.001	437±159	406±147	366±147	<0.001
Vegetables	288±148	307±141	286±133	0.910	320±156	326±149	304±136	0.105
Fruit	7±37	29±73	143±99	<0.001	7±39	17±55	133±104	<0.001
Meat	57±79	121±87	142±82	<0.001	77±100	144±92	162±95	<0.001
Dairy	0±3	1±17	42±76	<0.001	0±4	1±15	33±68	<0.001
Bean	39±58	91±70	104±66	<0.001	39±62	101±74	115±74	<0.001
Mets Markers								
Waist circumference	81±10	80±10	80±10	0.007	83±10	83±10	85±10	0.001
Serum triglyceride	132±103	127±90	126±90	0.220	137±117	155±149	167±171	0.001
HDL-C	58±17	57±14	58±14	0.600	55±15	55±18	51±16	0.001
Fasting blood glucose	95±22	93±19	92±14	0.076	96±22	96±24	96±25	0.448
Systolic blood pressure	121±17	119±17	119±16	0.032	124±16	124±16	122±15	0.210
Diastolic blood pressure	78±10	77±10	78±10	0.154	81±10	81±11	81±10	0.454
MetS^3^ (%)	19	17	16	0.236	9	11	14	0.031
Abdominal adiposity (%)	24	20	20	0.165	3	3	3	0.684
High serum triglyceride level (%)	26	26	25	0.562	28	34	35	0.060
Low HDL-C (%)	31	34	28	0.433	11	15	22	<0.001
Abnormal glucose homeostasis (%)	24	22	20	0.164	24	26	24	0.942
Elevated blood pressure (%)	34	27	28	0.085	41	40	39	0.657

1. Tertile cut-points of DDS are as follow: 1st, ≤ 3; 2nd, 4; 3rd, 5-6. Continuous variables are presented as mean ± SD and categorical variables are presented as percentages.

2. P trend was analyzed by chi-square trends analysis.

3. MetS was defined as the presence of three or more of the following components: (1) abdominal adiposity (WC ≥102 cm in men and ≥ 88 cm in women; (2) low serum HDL-cholesterol < 40 mg/dL for men and <50 mg/dL for women); (3) high serum triglyceride levels (<150 mg/dL); (4) elevated blood pressure (SBP ≥130mmHg or DBP ≥ 85 mmHg); and (5) abnormal glucose homeostasis (fasting plasma glucose level ≥100 mg/dL).

### Gender difference of MetS

**Figure 1:**
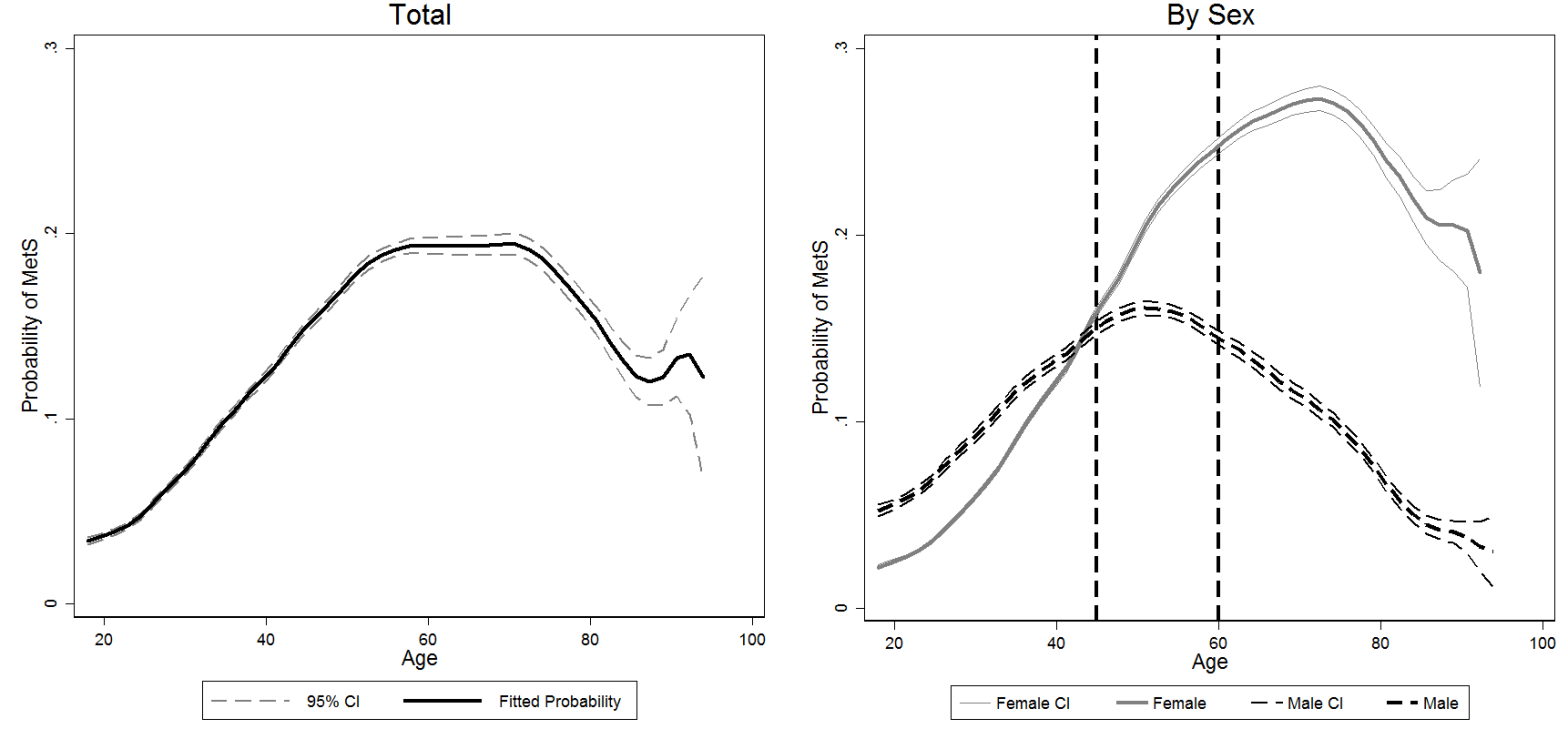
Association between predicted probability of having metabolic syndrome and age Left panel: association between predicted probability of having MetS and age in total population: the solid black line indicates the fitted probability of having metabolic syndrome at different age, the two gray dash lines indicate the 95% CI of fitted probability. Right panel: association between predicted probability of having MetS and age in female and male: the thick solid gray line indicates the fitted probability of having MetS at different age in female, and the two thin solid gray lines indicate the 95% CI of fitted probability of female; the thick dash black line indicates the fitted probability of having MetS at different age in male, and the two thin black dash lines indicate the 95% CI of fitted probability of male; The two vertical black dash lines divided participants into three groups: young: ≥18 & ≤45; adult: >45 & ≤60; old: >60). Adjusted for age, square of age, educational level (primary, middle and high), logarithm of income, smoking (yes/no), drinking (current drink or no), physical activity (light, moderate and heavy), localization (urban or rural; north or south), total energy intake and fat share. For total population regression, sex was also added as covariates.

**Figure 2:**
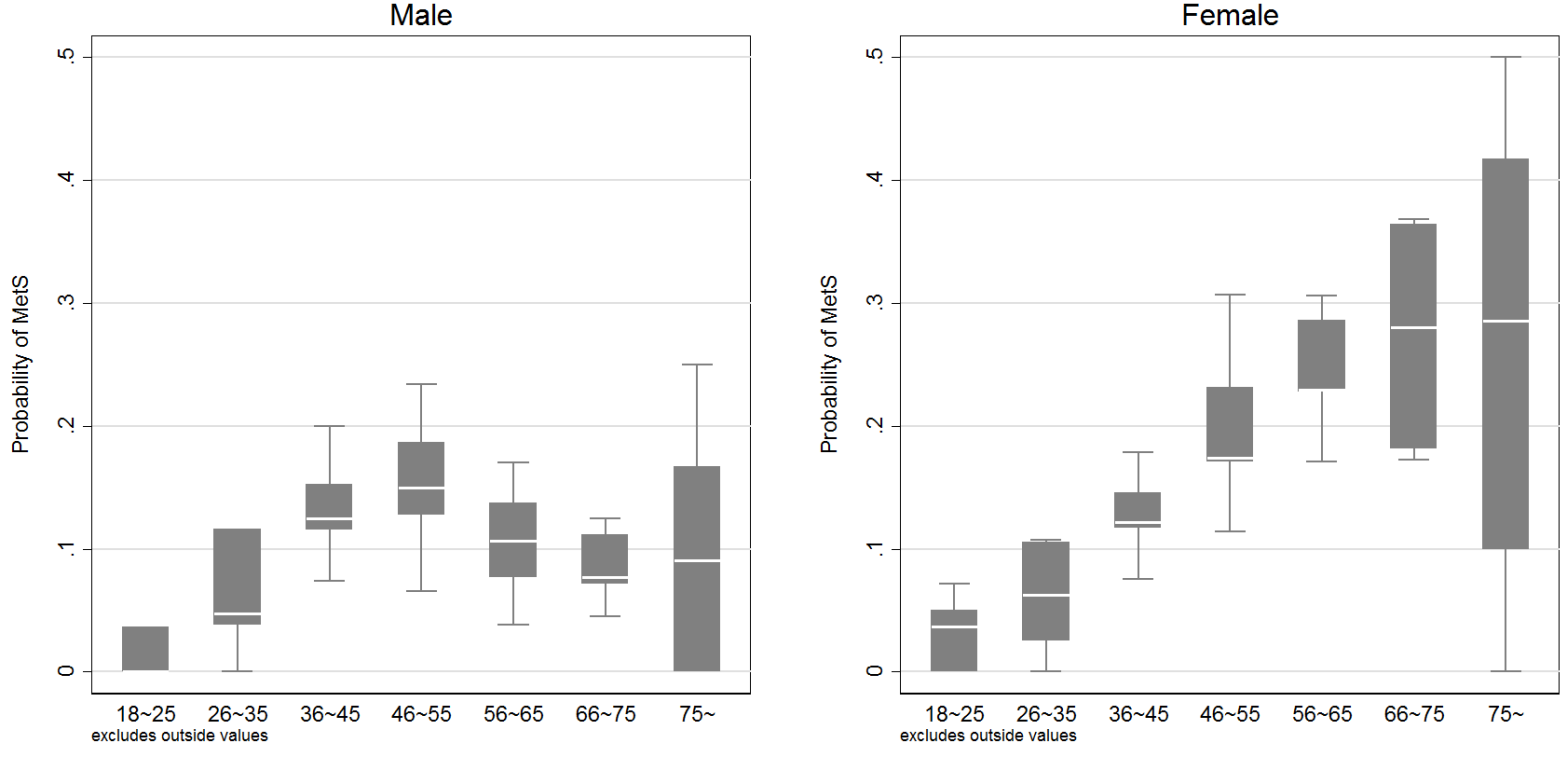
Real prevalence of having metabolic syndrome at each age group Left panel: prevalence of MetS at each age group for male. Right panel: prevalence of MetS at each age group for female. The Boxes are the 75th and 25th percentiles, and the white line within the box is the median value. Two gray horizontal lines refer to the upper and lower adjacent values. The upper adjacent value = 75th percentile+1.5×(75th percentile-25th percentile), and the lower adjacent value = 25th percentile-1.5×(75th percentile-25th percentile). Outside values, which are defined as those greater than the upper adjacent value or smaller than the lower adjacent value, are excluded from the box graph.

No significant association was detected between DDS tertiles and MetS when the analysis was done with the multivariable logistic regression in both male and female (See Table 3). However, female had significantly higher risk of being MetS (p<0.01). In addition, age and age square also significantly affected the likelihood of being MetS (all p<0.05), indicating the relationship between Mets and age was not linear. Figure 1 mapped the association between the predicted probability of MetS and age. An inverse U-shape was detected for all populations. In addition, different turning points were observed for female and male, that female reached the highest risk of MetS around 70 years old, while male exposed the highest risk around 50 year old. More importantly, female had lower risk of being MetS compared with their male counterparts before 43 years old, but they were more vulnerable to have MetS when the age was beyond 50 years old. In order to show the real prevalence of having MetS, we calculate the share of people having MetS at each age, and presented the results using box graph in Figure 2.

**Table 3:** Multivariable adjusted^1^ association between MetS at DDS tertiles

	Total population (*n* = 4308)	Female (*n* = 2348)	Male (*n* = 1960)
DDS1(referent)	1	1	1
DDS2(ORs)	1.081(0.876, 1.334)^2^	0.998(0.759, 1.287)	1.323(0.921, 1.899)
DDS3(ORs)	1.013(0.780, 1.315)	0.868(0.622, 1.210)	1.386(0.903, 2.128)
Female(ORs)	1.537(1.178, 2.004)		
Age(ORs)	1.154(1.107, 1.203)	1.181(1.113, 1.252)	1.152(1.080, 1.230)
Square of age(ORs)	0.999(0.998, 0.999)	0.999(0998, 0.999)	0.999(0.998, 0.999)

^1^ Adjusted for age, square of age, educational level (primary, middle and high), ln (income), smoking (current smoking or no), drinking (current drink or no) , physical activity (light, moderate and heavy), localization (urban or rural; north or south), total energy intake and fat share. For total population regression, sex was added in addition.

^2^ Values are ORs (95%CI) unless otherwise indicated.

### Association between DDS and MetS and its components

Further investigation was conducted to find out whether the association between DDS tertiles and MetS odds varies at different age. We divided age into three groups, young (≤45), adult (45∼60), and old (>60). Age was treated as categorical variables and the interaction term between age and DDS tertiles was used to replace age and age square in the former model (see Table 4). Overall, more diversified diet played a protective role in MetS for young female, while it increased the odds of MetS for old female and male adult. In particular, MetS odds was 50% lower in tertile 2 vs. tertile 1 [0.50(0.34, 0.74)] and 63% lower in tertile 3 vs. tertile 1 [0.37(0.22, 0.64)] for young female, while old female had 69% higher odds of MetS in the third tertile vs. first tertile [1.69(1.05, 2.72). Similarly, male adults had 59% and 118% higher odds of MetS in the second and third tertile vs. first tertile [1.59(1.04, 2.44); 2.18(1.30, 3.65)], individually.

**Table 4:** Multivariable^1^ adjusted analysis of the association between DDS and MetS and its components for each age group

Indices		Female				Male			
		DDS1	DDS2	DDS3	P trend	DDS1	DDS2	DDS3	P trend
MetS^2^	Young^3^	1	0.50(0.34, 0.74)^6^	0.37(0.22, 0.64)	0.000	1	1.17(0.73, 1.88)	1.05(0.59, 1.89)	0.731
	Adult^4^	1	1.31(0.96, 1.79)	1.02(0.68, 1.53)	0.564	1	1.59(1.04, 2.44)	2.18(1.30, 3.65)	0.002
	Old^5^	1	1.18(0.80, 1.76)	1.69(1.05, 2.72)	0.030	1	1.18(0.69, 2.02)	0.85(0.42, 1.73)	0.870
Component of metabolic syndrome									
High serum TGs	Young	1	0.64(0.47, 0.87)	0.52(0.35, 0.80)	0.000	1	1.25(0.93, 1.68)	1.20(0.82, 1.74)	0.214
	Adult	1	1.25(0.94, 1.66)	1.43(1.01, 2.01)	0.030	1	1.56(1.17, 2.08)	1.30(0.89, 1.91)	0.032
	Old	1	1.37(0.96, 1.95)	1.06 (0.67, 1.68)	0.364	1	0.71(0.47, 1.06)	0.71(0.44, 1.16)	0.076
Low HDL	Young	1	1.10(0.84, 1.44)	0.83(0.59, 1.17)	0.444	1	2.05(1.38, 3.30)	2.89(1.85, 4.52)	0.000
	Adult	1	1.24(0.95, 1.63)	0.82(0.58, 1.16)	0.624	1	1.42(0.95, 2.11)	2.00(1.24, 3.24)	0.004
	Old	1	1.05(0.74, 1.49)	0.74 (0.47, 1.17)	0.333	1	0.59(0.33, 1.06)	1.03(0.56, 1.90)	0.618
Abdominal adiposity	Young	1	0.49(0.34, 0.70)	0.46(0.29, 0.74)	0.000	1	0.74(0.30, 1.84)	0. 74(0.26, 2.14)	0.529
	Adult	1	1.20(0.89, 1.62)	1.03(0.70, 1.51)	0.621	1	1.29(0.61, 2. 74)	0.72(0.25, 2.10)	0.737
	Old	1	1.26(0.86, 1.86)	1.80(1.15, 2.82)	0.009	1	0.33(0.07, 1.46)	0.20(0.03, 1.54)	0.060
Elevated blood pressure	Young	1	0.41(0.30, 0.57)	0.26(0.17, 0.41)	0.000	1	0.49(0.36, 0.66)	0.51(0.34, 0.76)	0.000
	Adult	1	1.00(0.75, 1.31)	1.18(0.84, 1.65)	0.432	1	1.08(0.81, 1.43)	1.21(0.83, 1.77)	0.315
	Old	1	1.54(1.09, 2.19)	2. 62(1.67, 4.09)	0.000	1	2.49(1.76, 3.53)	1.54(1.00, 2.35)	0.000
Impaired fasting glucose	Young	1	0.47(0.33, 0.67)	0.37(0.23, 0.59)	0.000	1	0.71(0.50, 1.00)	0.63(0.41, 0.98)	0.018
	Adult	1	1.32(0.98, 1.78)	1.31(0.91, 1.90)	0.071	1	1.29(0.95, 1.75)	1.29(0.86, 1.94)	0.113
	Old	1	1.36(0.95, 1.96)	1.50(0.95, 2.38)	0.035	1	1.35(0.93, 1.97)	1.04(0.65, 1.68)	0.442

^1^ Adjusted for educational level (primary, middle and high), logarithm of income, smoking (yes/no), drinking (current drink or no) , physical activity (light, moderate and heavy), localization (urban or rural; north or south), total energy intake and fat share.

^2^ Metabolic syndrome was defined as the presence of three or more of the following components: (1) abdominal adiposity (WC ≥ 102 cm in men and ≥ 88 cm in women; (2) low serum HDL-cholesterol <40 mg/dL for men and <50 mg/dL for women); (3) high serum triglyceride levels (<150 mg/dL); (4) elevated blood pressure (SBP ≥130mmHg or DBP ≥ 85 mmHg); and (5)abnormal glucose homeostasis (fasting plasma glucose level ≥100 mg/dL).

^3^ Young is defined as ≤ 45 y

^4^ Adult is defined as <45 y and ≤ 60 y

^5^ Old is defined as <60 y

^6^ Values are ORs (95%CI) unless otherwise indicated.

Finally, the associations between five individual components of MetS were investigated separately in multivariable adjusted logistic regressions in both sexes and different categorical of ages (see Table 4). The lower probability of having MetS in the higher tertiles of DDS for young female could be attributed to the declining risk of high serum TGs, lower abdominal adiposity, and elevated blood pressure and impaired fasting glucose in higher DDS tertiles. On the contrary, the increasing risk of having MetS in the higher tertiles of DDS for old female could be attributed to the greater odds of abdominal adiposity and elevated blood pressure in tertile 2 or 3. Different from female, all MetS risk factors, except for abdominal adiposity, were significantly associated with DDS tertiles for male. In particular, a higher DDS was associated with increasing risk of having high serum triglyceride level and low HDL for male adult, and contributed to high odds of elevated blood pressure for old male. Intriguingly, a positive relationship was observed between DDS and low HDL-C in young men as well. However, a negative association between DDS and elevated blood pressure was detected; similar relationship was also found for abnormal fasting glucose.

To test the robustness of our findings, we also conducted sensitivity analysis by adding BMI in the multivariable adjusted logistic regressions. The main results were presented in Supplementary Tables 1 and 2, and the association between the probability of having metabolic syndrome and age was mapped in Supplementary Figure 1. Even the odds ratios changed a little bit, the main results are consistent with current findings.

## DISCUSSION

Two important findings were observed in the present study: first, female, especially those older than 50, had a higher risk of being MetS compared with their male counterparts; second, the association between DDS and MetS varies between men and women and across different ages. We found more diversified diet played a protective role in MetS for young female adults, but increased the risk of having MetS when women got older. Similar trends were observed in male as well, except the positive association between the HDL-C level and dietary diversity in young male.

This study presented that old women were more vulnerable to have MetS compared with men. Women’s risk of having MetS kept raising until 70 years old, while men’s risk turned down after 50 years old. Our results were in line with a current study which found that women had higher risk of being arterial stiffness than men [28]. The reason might be attributed to multi-factors, including sex-specific biology (different hormone status), disparate sex psychosocial stressors and lifestyle which influence the pathogenesis of MetS.

Different from most previous studies, which claimed that higher diversity dietary was the protective factor of being MetS [9, 21-23], we went deeper into this topic and found heterogeneous associations across different ages. The discrepancy might be attributed to the different foods consumption in the higher category of DDS. Previous studies declared that upper category of DDS was characterized by more healthy foods, such as vegetables, fruits and low fat dairy, which contain plant-based fibers, antioxidants and calcium but low in energy and glycemic index, and can regulat the blood glucose and pressure, played protective effect in MetS [29-33]. However, our data show that higher DDS was characterized with constant intake of vegetable, but more fruit, meat, dairy and bean. Although the average consumption of fruit was almost 20 times in the third tertile compared with the first tertile of DDS, significantly higher meat consumption was also detected in the third vs. first tertiles of DDS. Even the meat consumption keep rising and was always greater or equal to the fruit consumption at each DDS tertile, insufficient consumption of fruit but overconsumption of meat at higher tertile of DDS were detected since the recommended intake of fruit and meat for an adult was 200-500g and 40-75g respectively in Chinese Food Pagoda 2016 [34].Additionally, although the third tertile had the most consumption of dairy, the amount is far behind the recommendation level (300g/day) according to China Food Pagoda 2016. Hence, the protective effect of dairy may not appeared in MetS. The decreasing intake of carbohydrate at higher DDS tertile is consistent with the reduction in grain consumption. One possible reason for our findings would be that China is still a developing country during nutrition transition. Along with the economic development, food facilities and accessibility in each living community increased a lot, which diversified Chinese peoples’ diet[19]. Nonetheless, the proportion of each type of food still needs to be balanced. Our previous study found a positive association between DDS and obesity in Chinese male adult as well[20]. Obesity is the pivotal risk factor of metabolic related chronic diseases. Therefore, the finding in previous study was also consistent with the positive association between DDS and MetS risk in middle aged men [20].

Age is the crucial factor in the process of diseases, especially chronic diseases [35]. In our analysis, the middle age men and old women tend to be the most vulnerable group of having MetS. However, the risk declined when participants get old. A cohort study of ischemic heart disease demonstrated that smoking was the significant risk for initial coronary heart disease for men aged 35-64 but not for men aged 65-94, using data from 3983 Canadian Air Force male aircrew. They speculated a higher mortality rate among smokers at younger ages [36]. Additionally, the predicted propensities of having MetS of men and women both followed an inverse-U shape, and the risks reached their peak values around 70 years old for female and 50 years old for male respectively. One study using American heart association data also denoted that the average age of a first heart attack is 64.7 and 72.2 years old for men and women respectively [37]. The non-linear relationship between age, DDS and MetS could be attributed to two factors: lifestyle and genetic background. Traditionally, middle aged men are the bread-earner in Chinese families. They are under huge pressure both from work and family due to strong competition in promotion and high dependence ratio from children and parents. Studies found that stress and deprived of sleep for long time could induce chronic disease, such as obesity, hypertension and diabetes [38-40]. The situation could be improved after retirement, because they do not need to work anymore, and their children grow up while their parents might pass by. Restricted by the data availability, our present study could not include those factors which remain for further research. On the other hand, genetics variables might also play an important role in the risk of being MetS. Numbers of research indicated that Apolipoprotein E (APOE) [41], cholesteryl ester transfer protein (CETP) [42] and proinflammatory cytokines [43] are involved in the lipid metabolism. One recent research found that single nucleotide mutation (SNP) *rs3764261* in CETP combined with a Mediterranean diet for one year, the HDL-C level was enhanced and the concentration of triglycerides was reduced in the minor allele carrier [42]. In present study, HDL-C and triglycerides were also significantly correlated with DDS for middle aged men, which indicated that these two indicators are sensitive and malleable by age and diet.

Several limitations should be taken into account when implementing the results in future study. First, our study is based on a cross-sectional data, which prevents the causal inference between DDS and MetS. Second, intake of oil is an important factor in the process of MetS, and our data indicated that average intake of oil is higher than the recommended level according to Chinese Food Pagoda 2016 (unpublished data). However, individual oil consumption is not available in our dataset, which restricted our further analysis. Additionally, the inter-relations among individual components of MetS may attenuate the association between DDS and MetS. Finally, MetS is the result of jointly work of genetic and environmental factors. Ignoring these factors might bias our estimation of the association between DDS and MetS. Therefore, interventional or longitudinal study should be carried out to reinforce our analysis.

In spite of the limitations of our research, this study still has a number of strengths which could contribute to the current literature. First, we predicted the risk of having MetS for men and women at different ages, and found that women have lower risk of being MetS in comparison with men at young age, but the risk overpasses men when they get older. Moreover, middle-aged men and old women are more vulnerable to MetS, and the susceptible age of MetS for men and women are around 50 years old and 70 years old respectively. Thus, further intervention strategy should be design for men and women individually. Second, we exclude patients with hypertension, stork, diabetes and myocardial infarction, so the dietary of the remaining participants can better reflect the real food consumption in association analysis. Third, using a population based, represented sample of Chinese adults, our finding could be extrapolated to generalize Chinese adults.

MetS is considered as a substantial predictor of CVD and all-cause mortality. China is undergoing ageing and nutrition transition simultaneously and inevitably. MetS can be well controlled via health promotion and health education strategy, which can be designed specifically for each subpopulation. Regarding the ageing and shrinking population in China, improving the health status of ageing people is a long-term strategy for the labor force market [44], and we believe reducing the prevalence of MetS is a practical implementation.

## MATERIALS AND METHODS

### Population and data collection

CHNS was implemented by the University of North Carolina at Chapel Hill (UNC-CH) and the Chinese Institute of Nutrition and Food Safety (INFS), and China Center for Disease Control and Prevention (CCDC). This survey was approved by the Institutional Review Board of aforementioned institutions. All participants provided the written informed consent. Detailed information about this survey and laboratory examinations has been described elsewhere [45]. This study only extracted 2009 data because as the biochemical data was only available in 2009. Only adults (≥18 years old) with complete information on food consumption and biochemical data were included in our analysis (n=5118). We excluded women who were pregnant or lactating (n=41). To avoided the distortion of outlier, adults with implausible daily energy intake (>7000kcal or <520kcal) were censored (n=4). Meanwhile, adults who have a history of metabolic related disease, such as myocardial infarction (n=15), diabetes (n=90), and apoplexy (n=31) were excluded, because their diet might be changed after diagnostic disease. In addition, we also excluded people who take antihypertensive drugs (n=507). After matching food data with anthropometric data and biochemical data, individuals with incomplete information was removed (n=122). Finally, 4308 participants were included in the present study, of which 2348 are female and 1960 are male.

### Dietary assessment and dietary diversity score

Individual daily food consumption data were collected through face-to-face interviews for 3 consecutive days which are randomly distributed within one week. Trained field interviewers helped each participants recall the food that they consumed at home and outside in a 24-h period and recorded the codes (listed in the Food Composition Table of China), types, amounts, and locations of consumption for each food item by using food models and pictures. More detailed information about the dietary data in CHNS has been previously reported [45].

In line with previous studies, we combine the original 12 major food categories in Chinese Food Composition Table into 6 broad groups (grains, vegetables, fruits, meat/poultry/seafood, dairy, and beans/eggs/nuts) based on similarities in nutrient composition and dietary function. Detailed information about DDS has been depicted in previous studies [20, 46, 47]. Foods consumed less than a minimum amount (25g) per day was excluded to avoid measurement error caused by negligible consumption. Since the consumption of dairy was generally low in Chinese dietary, the cutoff point was adjusted to 10g per day. The DDS value ranges from 0 to 6 and higher value indicates greater diversity of diet [20].

### Outcome variables and covariates

Waist circumference, height and weight were measured by trained health worker using regularly calibrated equipments under the instructions of manufacturers. Blood pressure was measured after thrice rest in a seated position, each rest lasts for 5 minutes. It was utilized to calculate the average value of systolic blood pressure (SBP) and diastolic blood pressure (DBP). Blood sample was collected after 12-14h overnight fasting from all participants, and was stored in vacationer tubes. All blood samples were analyzed in the central laboratories of China-Japan Friendship Hospital. Fasting plasma glucose was measured by GOD-PAP, using the kit produced by Randox, UK. Serum high-density lipoprotein cholesterol (HDL-C) concentration was measured by enzymatic method; serum triglyceride levels was measured by CHOD-PAP; these two testing reagent were produced by the Kyowa, Japan. The last three indices were measured by the Hitachi 7600 machine.

MetS was defined according to the harmonized definition of the International Diabetes Federation and other organizations [1], that three or more out of five following criteria are considered as MetS: (1) central adiposity (WC >102 cm in men and >88cm in women); (2) serum HDL-C < 50 mg/dL in women or < 40 mg/dL in men; (3) serum triglyceride levels > 150 mg/dL; (4) SBP ≥ 130mm Hg or DBP ≥ 85mm Hg; (5) fasting plasma glucose ≥100 mg/dL.

Socio-economic characteristics such as sex, age, income per capita, educational level (Primary, middle and high), total energy intake, smoking status (0= currently not smoking, 1= currently smoking) and drinking status (0= did not drink in the past year, 1= drank alcohol in the past year), and residential area (urban and rural, north and south) were collected as co-variables. Physical activity was defined according to occupation (1=light physical activity, working in a sitting or standing position, such as office worker, teacher; 2=moderate physical activity, such as student or driver; 3= heavy physical activity, such as farmers, loader, miner), and adjusted in the model as well. Adults were categorized into three age groups according to the WHO standards (young: ≥18 & ≤45; adult: >45 & ≤60; old: >60).

### Statistical analysis

The descriptive statistical were presented at each tertiles of DDS for each sex(1st, ≤3; 2nd, 4; 3rd, 5-6). Continuous variates are presented as means ± SEs and categorical variables are presented as percentages.

The association between DDS and MetS and its individual components was detected by multivariable-adjusted logistic regression, both the OR values and 95% CI were presented. Aformentioned socio-economic variables were adjusted in the multivariable regression model. Income and total energy intake were measured in logarithm. The association between predicted probability of MetS and age was mapped using kernel weighted local polynomial smoothing; both the fitted probability and 95% confidence interval were presented. The linear trend of odds ratio for DDS was tested by taking DDS as continuous variable in the multivariable logistic regression.

All analyses were performed with Stata MP 13.0 (Stata Crop, USA) and P<0.05 was considered as statistical significance.

## SUPPLEMENTARY MATERIALS FIGURE AND TABLES



## Abbreviations

DDS: dietary diversity score
CVD: cardiovascular disease
T2D: type 2 diabetes
MetS: metabolic syndrome
HDL-C: high-density lipoprotein cholesterol
