# Supplementary material for: Gender difference of metabolic syndrome and its association with dietary diversity at different ages
Source: Oncotarget. 2017 Sep 2;8(43):73568–78. doi: 10.18632/oncotarget.20625 (PMC5650282; doi:10.18632/oncotarget.20625)
Supplement: Supplementary file 1 [file oncotarget-08-73568-s001.pdf]

Gender difference of metabolic syndrome and its association with dietary diversity at different ages

SUPPLEMENTARY MATERIALS

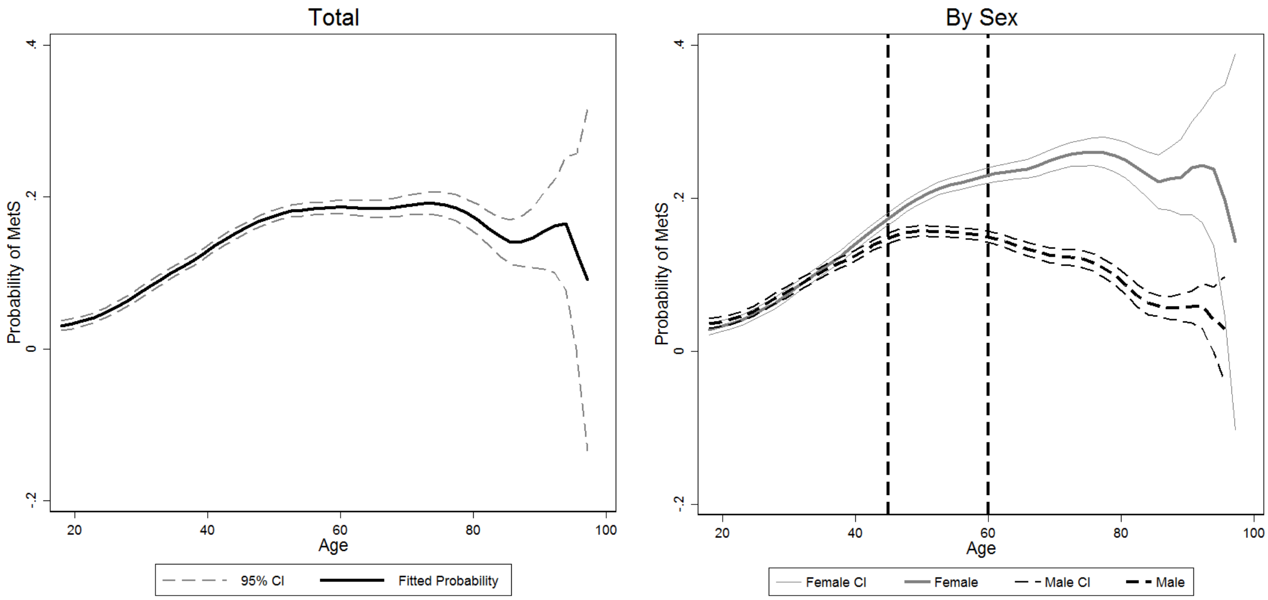

Supplementary Figure 1: Association between predicted probability of having metabolic syndrome and age with additional adjustment of BMI

Supplementary Table 1: Multivariable adjusted[1] Odds Ratio and 95% confidence interval of metabolic syndrome at three DDS tertiles

ORs	Total population (n=4308)	Female (n=2348)	Male (n=1960)
DDS1	1	1	1
DDS2	1.051(0.836, 1.320)[2]	0.971(0.721, 1.310)	1.248(0.856, 1.819)
DDS3	1.034(0.782, 1.366)	0.862(0.597, 1.247)	1.395(0.894, 2.176)
Female	1.664(1.250, 2.215)		
age	1.084(1.035, 1.136)	1.098(1.025, 1.176)	1.101(1.027, 1.180)
age square	0.999(0.999, 1.000)	1.000(0.999, 1.000)	0.999(0.998, 1.000)
BMI	1.354(1.312, 1.397)	1.387(1.330, 1.446)	1.295(1.233, 1.360)

[1] Adjusted for age, square of age, educational level (primary, middle and high), ln (income), smoking (current smoking or no), drinking (current drink or no) , physical activity (light, moderate and heavy), localization (urban or rural; north or south), BMI, total energy intake and fat share. For total population regression, sex was added in addition.
[2] Values are Ors (95%CI) unless otherwise indicated.

Supplementary Table 2: Multivariable adjusted[1] Odds Ratio and 95% confidence interval of MetS[2] and its risk factors at three DDS tertiles for each age group

Indices	ORs (95% CI)	Female				Male			
		DDS1	DDS2	DDS3	ptrend	DDS1	DDS2	DDS3	ptrend
MetS	Young[3]	1	0.54(0.36, 0.81)[6]	0.41(0.23, 0.72)	0.000	1	1.10(0.68, 1.78)	0.93(0.51, 1.70)	0.927
	Middle aged[4]	1	1.10(0.78, 1.56)	0.92(0.59, 1.44)	0.873	1	1.33(0.85, 2.08)	2.10(1.22, 3.62)	0.009
	Old[5]	1	1.38(0.87, 2.18)	1.69(0.98, 2.92)	0.040	1	1.34(0.76, 2.36)	1.08(0.53, 2.23)	0.562
Component of metabolic syndrome									
High serum TGs	Young	1	0.69(0.50, 0.94)	0.57(0.37, 0.86)	0.000	1	1.23(0.91, 1.67)	1.16(0.78, 1.73)	0.302
	Adult	1	1.09(0.81, 1.47)	1.34(0.94, 1.91)	0.128	1	1.36(1.01, 1.85)	1.21(0.81, 1.81)	0.153
	Old	1	1.46(1.01, 2.11)	0.98(0.61, 1.58)	0.484	1	0.72(0.48, 1.09)	0.80(0.48, 1.33)	0.192
Low HDL	Young	1	1.18(0.90, 1.56)	0.91(0.64, 1.28)	0.869	1	2.00(1.35, 2.98)	2.87(1.82, 4.53)	0.000
	Adult	1	1.11(0.84, 1.46)	0.75(0.53, 1.08)	0.255	1	1.24(0.82, 1.86)	1.87(1.14, 3.08)	0.017
	Old	1	1.09(0.75, 1.58)	0.68(0.42, 1.11)	0.250	1	0.61(0.34, 1.11)	1.17(0.63, 2.15)	0.887
Abdominal adiposity	Young	1	0.43(0.27, 0.68)	0.49(0.26, 0.91)	0.002	1	0.33(0.08, 1.42)	0.37(0.10, 1.42)	0.108
	Adult	1	0.91(0.61, 1.34)	0.88(0.56, 1.38)	0.538	1	1.06(0.41, 2.72)	0.64(0.12, 3.48)	0.661
	Old	1	1.79(1.06, 3.02)	2.12(1.21, 3.72)	0.003	1	0.50(0.07, 3.45)	0.92(0.13, 6.69)	0.690
Elevated blood pressure	Young	1	0.42(0.31, 0.58)	0.27(0.17, 0.42)	0.000	1	0.46(0.34, 0.63)	0.48(0.32, 0.71)	0.000
	Adult	1	0.87(0.65, 1.15)	1.10(0.77, 1.56)	0.887	1	0.96(0.71, 1.28)	1.14(0.77, 1.67)	0.662
	Old	1	1.65(1.15, 2.38)	2.57(1.60, 4.15)	0.000	1	2.64(1.84, 3.80)	1.70(1.11, 2.61)	0.000
Impaired fasting glucose	Young	1	0.50(0.35, 0.71)	0.39(0.24, 0.63)	0.000	1	0.69(0.49, 0.98)	0.61(0.39, 0.96)	0.013
	Adult	1	1.20(0.89, 1.62)	1.24(0.85, 1.81)	0.192	1	1.19(0.87, 1.62)	1.24(0.83, 1.86)	0.220
	Old	1	1.43(0.98, 2.07)	1.43(0.90, 2.28)	0.047	1	1.39(0.95, 2.02)	1.11(0.68, 1.80)	0.307

[1] Adjusted for educational level (primary, middle and high), logarithm of income, smoking (yes/no), drinking(current drink or no) , physical activity (light, moderate and heavy), localization (urban or rural; north or south), BMI, total energy intake and fat share.

[2] MetS was defined as the presence of three or more of the following components: (1) abdominal adiposity (WC ≥ 102 cm in men and ≥ 88 cm in women; (2) low serum HDL-cholesterol ＜40 mg/dL for men and ＜50 mg/dL for women); (3) high serum triglyceride levels (＞150 mg/dL); (4) elevated blood pressure (SBP ≥130mmHg or DBP ≥ 85 mmHg); and (5)abnormal glucose homeostasis (fasting plasma glucose level ≥100 mg/dL).

[3] Young is defined as ≤ 45 y.

[4] Adult is defined as ＞45 y and ≤ 60 y.

5 Old is defined as >60 y.

6 Values are ORs (95%CI) unless otherwise indicated.
